# Artificial Intelligence in Gynecologic Oncology: Current Applications, Clinical Challenges, and Future Perspectives

**DOI:** 10.7759/cureus.111703

**Published:** 2026-06-29

**Authors:** Dimitrios Alefragkis, George Mpourazanis, Pietro Serra, Stefanos Flindris, Rüediger Schulz-Wendtland, Fatma Goshi, Anna Papanikolaou, Petros Papalexis, Apostolos Ntanasis, Dimitra Bartzi, Romanos Vogiatzis, Antonio Simone Laganà, Ioannis Korkontzelos, Panagiotis Tsirkas

**Affiliations:** 1 Second Department of Critical Care, Attikon University General Hospital, Athens, GRC; 2 Department of Nursing, School of Health Sciences, National and Kapodistrian University of Athens, Athens, GRC; 3 Department of Obstetrics and Gynecology, General Hospital of Ioannina "G. Hatzikosta", Ioannina, GRC; 4 Unit of Obstetrics and Gynecology, University Hospital "Policlinico Paolo Giaccone", Palermo, ITA; 5 Department of Health Promotion, Mother and Child Care, Internal Medicine and Medical Specialties (PROMISE), University of Palermo, Palermo, ITA; 6 Second Department of Obstetrics and Gynecology, Hippokration General Hospital Thessaloniki, Thessaloniki, GRC; 7 Department of Obstetrics and Gynecology, University Breast Center for Franconia, University Hospital Erlangen, Friedrich-Alexander-Universität Erlangen-Nürnberg, Erlangen, DEU; 8 Gynecology Clinic, Klinika Gjinekologjike "Dr Fatma Goshi", Sarande, ALB; 9 Department of Obstetrics and Gynecology, Faculty of Medicine, University of Ioannina, Ioannina, GRC; 10 First Department of Internal Medicine, Unit of Endocrinology, Laiko General Hospital, National and Kapodistrian University of Athens, Athens, GRC; 11 Department of Anesthesiology, General Hospital of Ioannina "G. Hatzikosta", Ioannina, GRC; 12 Department of Oncology, 251 Air Force General Hospital, Athens, GRC; 13 Dermatological Center (DermaZentrum), Ingolstadt Krankenhaus, Ingolstadt, DEU; 14 Obstetrics and Gynecology Unit, Ospedali Riuniti Villa Sofia-Cervello, Palermo, ITA

**Keywords:** ai radiomics, artificial intelligence, artificial intelligence (ai), automated pap smear clarification, digital pathology, disease prediction, gynecologic cancers, gynecologic oncology, tumor type identification

## Abstract

Cervical, endometrial, ovarian, vulvar, vaginal, fallopian tube, and gestational trophoblastic neoplasia (GTN) are major gynecologic cancers that significantly impact women's health globally. In spite of progress in surgery, chemotherapy, radiotherapy, and targeted therapies, results are still variable, and timely diagnosis frequently proves challenging. Artificial intelligence (AI) has progressively taken advantage of, in digital pathological conditions risk prognostication for gynecological pathologies like endometrial and ovarian cancers, and automated Pap smear clarification for cervical cancer. Multi-platform methods combining clinical approaches and imaging data may help with prognostic assessments and personalized therapies. Nevertheless, major clinical information arises from single-center retrospective studies with minimal external authentication, and challenges like data heterogeneity, the lack of systematized protocols, ethical concerns, algorithmic bias, transparency, and workflow integration must be addressed in light of wide-ranging clinical and scientific approval.

## Introduction and background

Gynecological cancers encompass a wide range of malignancies. These cancers evolve in the female reproductive system and contribute to high rates of illness and fatality worldwide. The persisting occurrence of such malignancies underscores the consequential urgency for research related to primary diagnosis and the development of efficacious therapy intended to strengthen the health outcomes for women affected by these diseases. Research on gynecological cancers is essential for improving women's health and alleviating the health burden [1].

According to the literature, the epithelial tissue is the origin of gynecological cancers, specifically endometrial, ovarian, and cervical cancers. Tumors are categorized according to the different reproductive tract locations where they can form [2]. Conventional treatments for gynecologic tumors include radiation therapy, chemotherapy, surgery, and targeted therapy. The pathophysiology and treatment of gynecological cancers, particularly endometrial cancer, have seen significant advancements in the last decade; however, despite advancements, patient prognosis remains poor. Treatment outcomes are highly variable, reflecting the complex and personalized nature of managing these cancers [3,4]. The etiology of gynecological cancers differs, leading to various clinical manifestations. Improved knowledge has enhanced treatment options and management strategies for patients [5,6]. Individualized care is essential, as conventional diagnostic methods may fall short for aggressive or early disease subtypes. Tools for early diagnosis, personalized risk assessment, and therapy selection based on individual clinical and biological profiles are urgently required.

Machine learning (ML), a subset of artificial intelligence (AI), is emerging as a transformative technology for effectively analyzing complex medical data [7,8]. ML models show significant potential in gynecologic oncology, aiding in tumor type identification, automating Pap smears, predicting recurrence or metastasis, and discovering novel biomarkers from genetic data. AI has enabled computers to perform tasks requiring human intelligence, such as image processing. Over the past decade, the application of AI in cancer imaging has increased, with AI radiomics being developed to assist physicians in cancer treatment [9-12]. The foundation of algorithms has shifted from simple convolutional neural networks (CNNs) to advanced transformer-based architectures as a result of recent advancements in AI. This development highlights the need for detailed usage guidelines for computational pathology products to mitigate potential biases influenced by pre-scanning procedures that impact AI system performance [13,14].

This narrative review emphasizes the limitations of current data in the field of gynecologic oncology and explores prospective directions for clinical application. It concentrates on the existing evidence within the literature, particularly regarding the integration of ML algorithms in the clinical practices of gynecologic oncologists, aimed at enhancing their decision-making processes.

Methods

This narrative review synthesizes peer-reviewed literature on AI applications in gynecological cancers, including ovarian, vulvar and vagina, cervical, endometrial, fallopian tube, and gestational trophoblastic neoplasia (GTN). The review focused on AI applications in screening, diagnosis, prognostic assessment, treatment planning, surgical support, and follow-up.

A literature search was performed in PubMed/MEDLINE (Medical Literature Analysis and Retrieval System Online), Scopus, Embase, and Google Scholar. This search mainly included articles published in English between 2023 and 2025, while older landmark or highly relevant studies were also considered when necessary. The search terms included different combinations of “Artificial Intelligence”, “AI”, “Gynecological cancers”, “AI applications”, “machine learning”, “radiomics”, “cervical cancer”, “ovarian cancer”, “vulvar cancer AND vagina cancer”, “fallopian tube cancer”, and “gestational trophoblastic neoplasia”.

Original research articles, retrospective studies, literature reviews, and systematic reviews were considered for inclusion when they discussed AI-related applications in gynecological oncology. Additional articles were also identified by manually reviewing the reference lists of selected papers.

The findings were organized narratively according to cancer type and clinical application. No meta-analysis was conducted because the available studies differed substantially in terms of study design, AI models, data sources, outcomes, and clinical settings. As this article was designed as a narrative review rather than a systematic review, a Preferred Reporting Items for Systematic reviews and Meta-Analyses (PRISMA) flow diagram and formal risk-of-bias assessment were not performed. This represents a methodological limitation, although the search strategy, eligibility criteria, and narrative synthesis approach were described to improve transparency.

## Review

Overview of AI applications in gynecological oncology

In the modern era we live in, the rapid development and integration of new technologies have influenced the management of gynecological cancers. AI and related computational approaches are increasingly being investigated across different stages of cancer types, including diagnostic evaluation and prognostic modeling, in order to design the most effective and high-quality treatments. Research indicates that AI has significant potential in digital pathology, imaging, and predicting clinical outcomes through data-driven approaches [15]. The reliance on retrospective datasets from individual institutions, despite the technical performance of many published systems, highlights the need for standardized development pipelines and well-structured prospective studies to facilitate clinical tool development and adoption [16,17].

Cervical Cancer

Cervical cancer serves as a critical domain for the application of AI due to its diverse algorithms for cytological imaging, human papillomavirus (HPV) screening, and colposcopy. These algorithms assist medical professionals by automating the interpretation of digitally enhanced colposcopy, HPV screening, and Pap smears. These applications have shown positive results in reducing subjectivity and enhancing diagnostic consistency [18,19]. AI systems utilizing neural networks such as ResNet (Residual Network) and Visual Geometry Group (VGG) can identify high-grade lesions with performance equal to or surpassing that of skilled colposcopists, providing high discrimination accuracy and real-time visual guidance during exams [19,20]. In addition, computational frameworks can integrate optical, cytological, and clinical data with detection algorithms to support lesion assessment, lymph node evaluation, recurrence prediction, and treatment monitoring. These frameworks evaluate lymph node condition, assess recurrence likelihood, and monitor treatment failure risk as recommended by the multidisciplinary team. They assist the team by improving monitoring strategies and facilitating the receipt and delivery of recommended adjuvant therapy [16,20]. In radiotherapy treatment planning, AI offers capabilities through previous contouring algorithms that facilitate consistent delineation of target tissues and organs at increased risk. Additionally, tools that optimize algorithms improve planning efficiency and dosimetric quality. However, efforts must be made to further validate these algorithms in order to confirm their clinical validity and benefits [16,17].

Endometrial Cancer

In endometrial cancer, AI applications are increasingly helpful because women presenting with abnormal uterine bleeding can be evaluated through computational techniques. Models have been developed that integrate clinical variables from patients with suspicious ultrasound findings. These give physicians the ability to estimate the probability of endometrial carcinoma or atypical hyperplasia, by providing immediate triage and prioritization of requirements for optimal interventional evaluation [21,16]. Furthermore, it has been shown that integration and application of radio mechanics to ultrasound further enhances the ability to differentiate benign from malignant endometrial pathology [22]. Another application of algorithms constitutes a central application in the preoperative stage. In particular, models that utilize ML algorithms can, in combination with data obtained from MRI, act as assistants for doctors. The algorithms' applications provide assessment and information regarding the depth of myometrial penetration, the degree of malignancy, lymph vascular involvement, and the detection of possible lymph node metastases. These are all well-established critical factors in designing an optimal surgical plan regarding the determination of surgical extent. These algorithms provide precision and predict the need for adjuvant therapies [16,19]. Overall, algorithms that combine imaging findings with clinical and pathological patient data are very promising in determining the assessment of therapeutic response and predicting survival, although further applications are required before their integration into daily care [23,24].

Ovarian Cancer

Ovarian cancer has received significant attention in recent years due to the difficulty physicians encounter in early diagnosis of the disease and the importance of targeted preoperative risk assessment. Recent work using ultrasound and MRI has shown that computational analysis can help differentiate benign from malignant adnexal lesions. Researchers have used information derived from ultrasound and MRI to train computational models capable of helping clinicians judge whether an adnexal mass is benign or malignant. In many cases, these models perform on par with specialists and act as an additional aid to frameworks already in use, such as International Ovarian Tumour Analysis (IOTA) and Ovarian-Adnexal Reporting and Data System (O-RADS) [22,25]. However, the heterogeneity in imaging workflows and methods of extracting results continues to be a challenge in determining whether they can be reproduced and adopted in various healthcare settings worldwide.

Computational radio genomics is emerging as the most crucial tool for understanding high-grade serous carcinoma [16,22]. Some recent studies have shown that certain imaging findings may be linked to the biological features of ovarian tumors. For example, certain patterns sometimes appear on scans of tumors with a specific type of genetic change or with problems in a process called homologous recombination. This information can be useful for the medical team in choosing the best treatment for their patients [16,26]. Other research groups have taken a broader approach, combining imaging results with routine blood tests and clinical observations. The aim is to work out whether it is feasible to remove as much of the tumor bulk as possible. This helps the oncology team decide whether the patient should have surgery first or start with chemotherapy to prepare them for surgery [15,24]. People are also interested in trying to work out how soon or often resistance to platinum-based therapy might appear, and in trying to predict what the long-term results will be. While these computer methods offer useful insights, they still need to be further tested before they can be used routinely [23,26].

In advanced ovarian cancer, AI also has an important role in treatment planning, particularly in the preoperative assessment of tumor burden and the likelihood of optimal cytoreduction [15,16]. Predictive models that combine image finding, clinical parameters, tumor markers, and performance status can contribute to the estimation of surgical feasibility and treatment response [23-26]. These tools can support multidisciplinary decision making regarding primary debulking surgery or neo-adjuvant chemotherapy, while the final treatment decision should remain based on expert clinical and surgical judgment [15,24].

Vulvar and Vaginal Cancer

There is not much data available on using computers to treat vulvar and vaginal cancers. This is partly because these cancers are rare and partly because it is hard to find large sets of data that are already collected [15,27]. Early explorations include using computers to automatically analyze photos and colposcopic images. Computer-generated image analysis facilitates standardizing the evaluation of suspicious lesions [15,20]. Digital pathology efforts have used neural network methods to analyze whole slide images to identify precancerous, inflammatory, and invasive lesions, although most studies are small-scale investigations in order to test the principal idea [28,29].

Because vulvar and vaginal cancers are rare, the development of reliable AI models is more challenging compared to other common gynecological malignancies [15,27]. Small datasets limit model training, while differences in lesion appearance, clinical image quality, colposcopic assessment, biopsy practices, and histopathological interpretation may affect reproducibility [20,28]. AI may also be useful in these malignancies by supporting the standardized assessment of suspicious lesions, assisting in the clarification of premalignant and malignant changes, and helping clinicians identify difficult cases that require biopsy or specialist referral. Future progress will depend on multicenter collaboration, shared image repositories, and external validation models specifically designed for rare gynecological malignancies [15,27].

Fallopian Tube Cancer

One more type of gynecologic cancer is fallopian tube cancer. Primary fallopian tube cancer is a rare gynecological malignancy, which makes precise preoperative differentiation challenging because it presents imaging and clinical similarities to ovarian cancer. Imaging methods such as ultrasound, MRI, and positron emission tomography-computed tomography (PET-CT) remain vital for evaluating suspicious fallopian tube lesions. Nevertheless, recent studies emphasize the necessity for more advanced diagnostic tools in this area [30].

While AI applications for fallopian tube cancer are limited due to its rarity, more advanced methods such as radiomics and ML classifiers can assist in assessing ovarian components and distinguishing whether the disease originates from the fallopian tubes or ovaries [26,31]. In pathology, AI algorithms that analyze whole slide images show promising results in detecting serous endometrial cancer of the fallopian tubes, a precursor lesion increasingly recognized in the development of high-grade serous carcinoma. Studies have also shown that AI models using deep learning algorithms can recognize subtle morphological abnormalities and p53 patterns that may not be detected during routine examinations [28,29]. Despite the progress that has been made, the current evidence is preliminary, underscoring the need to apply these algorithms to larger datasets and external validation before AI is adopted as routine practice in ovarian cancer diagnosis [16,17].

The role of AI in fallopian tube cancer may become more relevant as the relationship between the fallopian tube and high-grade serous carcinoma is increasingly recognized. AI-based pathology tools may assist in the detection of subtle precursor lesions, including serous tubal intraepithelial carcinoma, and can help in reducing interobserver variability in challenging histopathological cases [28,29]. In imaging, radiomics, and ML approaches may be useful in the differentiation between primary fallopian tube disease and ovarian involvement, although this remains a challenge due to overlapping clinical and radiological features [26,30,31]. At present, the available evidence remains preliminary, and larger datasets are required before AI can be considered a reliable tool in this rare malignancy [16,17].

Gestational Trophoblastic Neoplasia

As far as GTN is concerned, computational modeling is still in its early stages. Researchers have utilized different types of medical data to determine how well treatments might work for each individual patient. These types include features such as disease presentation and appearance, scan images, and how human chorionic gonadotropin (hCG) hormone levels change over time [20,27]. Some models have been designed for the prediction of methotrexate resistance, enabling doctors to consider alternative treatment options for high-risk patients early on [23,27]. However, the current research is limited by small sample sizes and the lack of multicenter validation [15,17].

In GTN, AI has the potential to refine risk stratification beyond conventional clinical scoring systems, particularly by integrating clinical characteristics, image findings, metastatic burden, prior pregnancy history, and serial hCG trends [20,23]. ML models can support the estimation of treatment resistance or relapse risk, which is clinically relevant for identifying patients who require closer monitoring or earlier treatment escalation [23,27]. GTN is uncommon, and outcomes are generally favorable when managed appropriately; these models require careful validation before clinical use to avoid overtreatment or unnecessary intervention [15,17]. The main disease-specific applications of AI in gynecologic oncology are summarized in Table 1. 

**Table 1:** Summary of AI applications by gynecologic cancer type. References: [15–17,20,22–31] HPV: human papillomavirus; IOTA: International Ovarian Tumour Analysis; O-RADS: Ovarian-Adnexal Reporting and Data System; AI: artificial intelligence

Cancer type	AI applications	Current evidence and limitations of AI use in clinical practice
Cervical cancer	Automated Pap smear interpretation, HPV screening support, colposcopy image analysis, lesion classification, lymph node and recurrence prediction, radiotherapy planning	One of the most developed areas of AI use in gynecologic oncology. However, external validation and standardized imaging protocols remain necessary before routine clinical use.
Endometrial cancer	Risk assessment in abnormal uterine bleeding, ultrasound-based malignancy prediction, MRI-based evaluation of myometrial invasion, lymphovascular invasion and nodal metastasis prediction, survival modeling	AI may support early triage and preoperative planning. Current evidence is promising but limited by retrospective datasets and heterogeneous model development.
Ovarian cancer	Differentiation of benign and malignant adnexal masses, integration with IOTA and O-RADS, radiomics and radiogenomics, cytoreduction feasibility prediction, platinum resistance, and survival prediction	AI may improve preoperative risk stratification and treatment planning. Reproducibility, imaging heterogeneity, and lack of multicenter validation remain major barriers.
Vulvar and vaginal cancer	Image-based lesion assessment, colposcopic image analysis, digital pathology, and histopathologic characterization	Evidence remains limited because of disease rarity and small datasets. Current applications are preliminary and require larger collaborative studies.
Fallopian tube cancer	Imaging-based differentiation from ovarian cancer, radiomics, digital pathology, and detection of precursor lesions such as serous tubal intraepithelial carcinoma	AI evidence is scarce due to the rarity and diagnostic overlap with ovarian cancer. Further external validation is required.
Gestational trophoblastic neoplasia	Prediction of methotrexate resistance, hCG trajectory modeling, treatment response assessment, multimodal risk stratification	AI may assist in the early identification of high-risk patients, but available evidence is early-stage and based on small samples.

Cross-cutting AI technologies in gynecologic oncology

Computational technologies now influence multiple aspects of gynecological oncology, extending beyond applications specific to individual diseases. Digitization is a particular area of progress, as whole-slide imaging combined with AI algorithms supports automated tissue evaluation, subtype characterization, and detection of subtle patterns relevant to prognosis [28,29]. Its widespread clinical adoption will require standardized staining and scanning procedures, as well as robust external validation, in order to be utilized by the multidisciplinary team as yet another tool [32]. Radiomics is another cross-disciplinary innovation. More specifically, by extracting high-dimensional quantitative features from imaging methods such as ultrasound, CT, MRI, and PET, radiomic models can help characterize tumor heterogeneity and predict recurrence or response to treatment in cervical, endometrial, and ovarian cancers [16,26]. Scanner variability and limited prospective validation are challenges that continue to limit scalability [17]. There is also growing interest in the integration of multipleomic data, where genomic, transcriptomic, proteomic, and imaging data are combined into unified prognostic frameworks. The principal cross-cutting AI technologies used across gynecologic oncology are presented in Table 2. 

**Table 2:** Cross-cutting AI technologies in gynecologic oncology References: [15–17,27–29,31–33]. AI: artificial intelligence

AI technology	Role across the care pathway	Added value
Machine learning	Risk prediction, therapy response, and survival modeling	Supports clinical decision-making using structured patient data
Deep learning	Cytology, pathology, ultrasoubd, MRI, CT, and PET image analysis	Improves automated detection and classification
Radiomics	Quantitative feature extraction from medical imaging	Helps characterize tumor heterogeneity and predict outcomes
Radiogenomics	Integration of imaging and molecular/genomic datas	Supports precision oncology and personalized therapy
Digital pathology	Whole-slide image analysis and histologic classification	Assists pathologists and may improve diagnostic consistency
Multimodal AI	Combination of clinical, imaging, pathology, and molecular data	Provides more individualized prognostic assessment
AI in Radiotherapy	Automated contouring, dose prediction, and therapy optimization	Improves planning efficiency and consistency
AI in Surgical Operations	Preoperative mapping, robotic support, and complication prediction	May improve surgical planning and intraoperative decision-making

AI systems also need to demonstrate safety, reproducibility, and clear clinical benefit before broader adoption [33]. Therefore, AI tools should be prospectively validated, clinically interpretable, and integrated into multidisciplinary decision-making rather than used as independent decision-markers [15,27]. Successful implementation will require standardized protocols, ethical oversight, and adaptation to real-world clinical settings. These AI tools can improve risk assessment and guide the medical team in choosing the optimal precision treatment, although large data sets from studies are still required for their adoption [16,26]. Also, in acrone therapy, AI applications are beginning to improve their design by supporting automated segmentation, dose distribution prediction, and treatment plan optimization, improving efficacy despite the limited trials that exist [16-17]. In the surgical procedures, algorithms are being investigated for preoperative mapping and guidance of the surgical team during surgery, using robotic and augmented reality systems, whose main purpose is to improve navigation of the surgeon and predict pre and postoperative complications [15,33]. In public health, AI acts as an aid to preventive population screening because it automates several procedures, such as the interpretation of Pap smears, HPV tests, and ultrasound examinations, which is important in resource-limited settings. The integration of tools that utilize deep learning and ML algorithms and their connection with data drawn from the population's electronic records can help even more in prevention and other public health issues, but there are still several critical points related to ethical and transparency issues that need to be resolved [27,32].

Ethical, technical, and regulatory challenges

Despite the increasing use of AI in gynecological oncology, several barriers still limit its routine clinical implementation. Many available models are based on retrospective or single-center datasets, which reduces their generalizability across different populations and healthcare systems globally [15,17]. This issue is mainly important in AI applications where validation across different clinical settings remains limited [27]. Data heterogeneity also affects reproducibility, especially when imaging protocols, scanner characteristics, radiomics workflows, pathology procedures, clinical documentation, and outcome definitions differ between studies [16,32]. Ethical and regulatory issues are equally important. Algorithmic bias, limited transparency, insufficient explainability, and data privacy concerns may reduce clinician trust and affect patient safety [27,32]. In surgical and treatment-planning setting workflows [16,32,33].

The main barriers to clinical implementation and potential strategies to address them are summarized in Table 3.

**Table 3:** Barriers to clinical implementation and proposed solutions References: [15–17,27,32,33] AI: artificial intelligence

Barrier	Clinical impact	Proposed solution
Data heterogeneity	Reduces reproducibility across centers	Standardized data collection and reporting protocols
Single-center retrospective studies	Limits generalizability	Multicenter prospective validation
Limited external validation	Weakens clinical reliability	Independent validation in different populations
Small datasets in rare cancers	Restricts model development	International registries and collaborative datasets
Lack of explainability	Reduces clinician trust	Explainable and transparent AI models
Bias and inequitable datasets	May worsen disparities	Diverse and representative training datasets
Ethical and privacy concerns	Delays clinical adoption	Strong governance and secure data handling
Workflow integration issues	Limits routine use	Clinician-friendly tools integrated into existing pathways

Future perspectives

As the years go by, it becomes increasingly clear that we need to come up with common standards for the data used in AI. If systems are trained on larger and more diverse populations, both geographically and biologically, models will perform more fairly and with greater reliability [17,27]. At the same time, personalized oncology is expected to become even more popular, as multimodal models and tools such as digital twins provide the ability to tailor treatment more directly and accurately to the needs of each individual patient [16,25]. Strong oversight and full transparency in how the models are developed and how they behave in clinical practice are required in order for these new tools to be adopted in daily clinical practice. Therefore, these are the cornerstones because the decisions made by the interdisciplinary team must provide absolute security. It is absolutely clear and obvious that the central axis of everything is the patient [32,33].

## Conclusions

AI is a rapidly developing and promising technology for possible decision-making for clinical practice in the near future. Many potentials are used in screening, diagnosis, prognostication, treatment planning, surgical guidance, and postoperative monitoring. AI systems may improve clinical decision-making and support individualized patient care, from digital pathology and multimodal predictive modeling, especially in automated Pap smear interpretation and radiomic tumor characterization. Despite the promising technical performance of AI in gynecologic cancer care, most supporting evidence is preliminary and derived from limited single-institution studies. Key challenges such as data heterogeneity, limited external validation, and regulatory issues need to be addressed before broader clinical application. Future progress requires the development of explainable AI models, large multicenter studies, standardized data frameworks, and equitable training datasets. AI should augment, not replace, clinical expertise, prioritizing patient-centered approaches to improve safety and individualized outcomes in treatment.

## References

[1] Vyas A, Kumar K, Sharma A, Verma D, Bhatia D, Wahi N, Yadav AK (2025). Advancing the frontier of artificial intelligence on emerging technologies to redefine cancer diagnosis and care. Comput Biol Med.

[2] Georgakopoulos P, Mehmood S, Akalin A, Shroyer KR (2012). Immunohistochemical localization of HE4 in benign, borderline, and malignant lesions of the ovary. Int J Gynecol Pathol.

[3] Bjurberg M, Holmberg E, Borgfeldt C (2019). Primary treatment patterns and survival of cervical cancer in Sweden: a population-based Swedish Gynecologic Cancer Group Study. Gynecol Oncol.

[4] Gupta S, Maheshwari A, Parab P (2018). Neoadjuvant chemotherapy followed by radical surgery versus concomitant chemotherapy and radiotherapy in patients with stage IB2, IIA, or IIB squamous cervicalcancer: a randomized controlled trial. J Clin Oncol.

[5] Besharat AR, Giannini A, Caserta D (2023). Pathogenesis and treatments of endometrial carcinoma. Clin Exp Obstet Gynecol.

[6] Zhang C, Sheng Y, Sun X, Wang Y (2023). New insights for gynecological cancer therapies: from molecular mechanisms and clinical evidence to future directions. Cancer Metastasis Rev.

[7] Zhang B, Shi H, Wang H (2023). Machine learning and AI in cancer prognosis, prediction, and treatment selection:a critical approach. J Multidiscip Healthc.

[8] Öznacar T, Güler T (2025). Prediction of early diagnosis in ovarian cancer patients using machine learning approaches with Boruta and advanced feature selection. Life (Basel).

[9] Syrou N, Mpourazanis G, Tsirkas P, Valamontes A, Adamopoulou J (2025). Clinical data analysis using machine learning algorithms to predict the progression of type 2 diabetes. Mesopot J Artif Intell Healthcar.

[10] Alefragkis D, Chougia V (2025). The role of artificial intelligence in cardiothoracic surgery: a narrative review. Prog Health Sci.

[11] Bi WL, Hosny A, Schabath MB (2019). Artificial intelligence in cancer imaging: clinical challenges and applications. CA Cancer J Clin.

[12] Mysona DP, Kapp DS, Rohatgi A (2021). Applying artificial intelligence to gynecologic oncology: a review. Obstet Gynecol Surv.

[13] Ersavas T, Smith MA, Mattick JS (2024). Novel applications of convolutional neural networks in the age oftransformers. Sci Rep.

[14] Shah M, Polónia A, Curado M, Vale J, Janowczyk A, Eloy C (2025). Impact of tissue thickness on computationalquantification of features in whole slide images for diagnostic pathology. Endocr Pathol.

[15] Restaino S, De Giorgio MR, Pellecchia G (2025). Artificial intelligence in gynecological oncology fromdiagnosis to surgery. Cancers (Basel).

[16] Bai G, Huo S, Wang G, Tian S (2025). Artificial intelligence radiomics in the diagnosis, treatment, and prognosis of gynecological cancer: a literature review. Transl Cancer Res.

[17] Zhang Y, Qin Q (2025). Prospects and challenges of deep learning in gynecologic malignancies. Front Oncol.

[18] Taddese AA, Tilahun BC, Awoke T, Atnafu A, Mamuye A, Mengiste SA (2023). Deep-learning models for image-based gynecological cancer diagnosis: a systematic review and meta- analysis. Front Oncol.

[19] Swarnkar B, Khare N, Gyanchandani M (2025). The evolving role of deep learning in image-based gynecological cancer diagnosis: a comprehensive review. Array.

[20] Wang Q, Zhou X, Wu J, Miao W, Shen B, Shi R (2025). Research progress of artificial intelligence in the early screening, diagnosis, precise treatment and prognosis prediction of three central gynecological malignancies. Front Oncol.

[21] Li YX, Lu Y, Song ZM, Shen YT, Lu W, Ren M (2025). Ultrasound-based machine learning model to predict the risk of endometrial cancer among postmenopausal women. BMC Med Imaging.

[22] Moro F, Ciancia M, Sciuto M (2025). Performance of radiomics analysis in ultrasound imaging for differentiating benign from malignant adnexal masses: a systematic review and meta-analysis. Acta Obstet Gynecol Scand.

[23] Garg P, Krishna M, Kulkarni P, Horne D, Salgia R, Singhal SS (2025). Machine learning models for predicting gynecological cancers: advances, challenges, and future directions. Cancers (Basel).

[24] Paiboonborirak C, Abu-Rustum NR, Wilailak S (2025). Artificial intelligence in the diagnosis and management of gynecologic cancer. Int J Gynaecol Obstet.

[25] Zeng S, Jia H, Zhang H (2025). Multimodal ultrasound-based radiomics and deep learning for differential diagnosis of O-RADS 4-5 adnexal masses. Cancer Imaging.

[26] Huang Y, Peng W, Yang H (2025). Radiomics and radiogenomics in ovarian cancer: a review with a focus on ultrasound applications. Cancer Imaging.

[27] Akazawa M, Hashimoto K (2021). Artificial intelligence in gynecologic cancers: Current status and future challenges - a systematic review. Artif Intell Med.

[28] Asaturova A, Pinto J, Polonia A, Karpulevich E, Mattias-Guiu X, Eloy C (2025). Artificial intelligence tools for supporting histopathologic and molecular characterization of gynecological cancers: a review. J Clin Med.

[29] Joshua A, Allen KE, Orsi NM (2025). An overview of artificial intelligence in gynaecological pathology diagnostics. Cancers (Basel).

[30] Iacobellis G, Leggio A, Salzillo C, Imparato A, Marzullo A (2025). The imaging of primary fallopian tube carcinoma: a literature review. Cancers (Basel).

[31] Shrestha P, Poudyal B, Yadollahi S (2022). A systematic review on the use of artificial intelligence in gynecologic imaging - background, state of the art, and future directions. Gynecol Oncol.

[32] Fiste O, Liontos M, Zagouri F, Stamatakos G, Dimopoulos MA (2022). Machine learning applications in gynecological cancer: a critical review. Crit Rev Oncol Hematol.

[33] Dou Q, Nyangoh-Timoh K, Jannin P, Shen Y (2025). Artificial intelligence in gynecology surgery: current status, challenges, and future opportunities. Chin Med J (Engl).

